# Integrative Disulfidptosis‐Based Risk Assessment for Prognostic Stratification and Immune Profiling in Glioma

**DOI:** 10.1111/jcmm.70429

**Published:** 2025-02-24

**Authors:** Xiaowang Niu, Guangzhao Li, Ulf D. Kahlert, Leili Ding, Jing Zheng, Chen Li, Wenjie Shi, Lifen Huang, Zhengquan Yu

**Affiliations:** ^1^ Department of Neurosurgery The First Affiliated Hospital of Soochow University Suzhou China; ^2^ Department of Neurosurgery Suqian Hospital Affiliated to Xuzhou Medical University Suqian China; ^3^ Department of Neurosurgery Hefei First People's Hospital Hefei China; ^4^ Molecular and Experimental Surgery, Clinic for General‐, Visceral ‐, Vascular‐ and Transplantation Surgery, University Hospital Magdeburg Otto‐von‐Guericke University Magdeburg Germany; ^5^ Nantong University Nantong China; ^6^ Department of Pharmacy The First Affiliated Hospital of Guangxi Medical University Nanning China; ^7^ Clinicopathological Diagnosis & Research Center The Affiliated Hospital of Youjiang Medical University for Nationalities Baise China; ^8^ Key Laboratory of Tumor Molecular Pathology of Guangxi Higher Education Institutes Baise China

**Keywords:** disulfidptosis, glioma, immune microenvironment, prognosis, risk score

## Abstract

Disulfidptosis, a new form of programmed cell death, plays a role in multiple types of cancer. However, research on disulfidptosis in glioma is lacking. A disulfidptosis‐associated risk score was constructed using Cox regression modelling, while LASSO regression was applied for feature selection. To explore the relationship between the risk score and the immune microenvironment, we employed CIBERSORT, ssGSEA and ESTIMATE algorithms. Additionally, wet lab experiments were conducted to validate the functional role of the key disulfidptosis gene RPN1, demonstrating its ability to promote glioma cell proliferation and migration. Disulfidptosis genes were significantly upregulated in gliomas, influencing clinical features and survival. The risk score effectively predicted OS and varied among clinical subgroups. High‐risk scores correlated with tumour growth, invasion and immunosuppression. Patients with different risk scores showed distinct immune cell infiltration patterns. Most immune checkpoints and chemokines were positively associated with risk scores. Laboratory findings confirmed that RPN1 significantly promoted glioma cell proliferation and migration. Disulfidptosis‐based risk assessment stratifies glioma prognosis and reveals immune microenvironment characteristics, offering insights for personalised treatment strategies.

## 1 Introduction

Disulfidptosis is a form of cell death induced by disulfide stress, occurring when glucose deprivation disrupts NADPH production [1, 2]. Recent studies appear to indicate that this disulfidptosis is the result of F‐actin's contraction and detachment from the plasma membrane, which is caused by an abnormal disulfide bond of the Actin cytoskeleton [3, 4, 5]. Inability to avert cell death caused by glucose deprivation has been observed for ferroptosis, apoptosis, cell necrosis and autophagy inhibitors, whereas using the disulfide stress‐reducing agent completely eliminates cell death [3]. As a novel type of programmed cell death (PCD), disulfidptosis may serve as a possible treatment for tumours [5].

An innovative approach to combating tumours is to regulate disulfidptosis to trigger tumour cell death. Recent bioinformatics analysis of high‐throughput sequencing has revealed that disulfidptosis has a multifaceted influence on the emergence, progression and anti‐tumour immunity of hepatocellular carcinoma [6], breast cancer [7], thyroid cancer [8], renal cell carcinoma [9] and bladder cancer [10]. Overexpression of SLC7A11 is a common occurrence in numerous cancers, including Glioblastoma (GBM) [11, 12]. Previous research has demonstrated that SLC7A11 is expressed at higher levels in GBM. When glucose was limited, the overexpression of SLC7A11 could lead to an increase in reactive oxygen species (ROS) and cell death. This cell death is believed to be a result of the effect of cell density on the breakdown of System Xc‐ protein by Lysosome [13]. Under glucose‐limited conditions, EGF has been shown to increase SLC7A11 expression in an mTOR‐dependent manner, leading to GBM cell death. The overexpression of SLC7A11 caused a reduction in the expression of the mismatch repair gene, resulting in higher levels of DNA repair double‐strand breaks (DSB) and improved sensitivity to radiotherapy [11]. However, glioma cell death under glucose‐restricted conditions may be linked to disulfidptosis, making it essential to investigate disulfidptosis in glioma for inducing cell death and discovering new therapeutic targets.

Currently, the predictive significance of disulfidptosis in glioma and its impact on the immune microenvironment remain unexplored. This study assessed the predictive significance of a risk score linked to disulfidptosis genes in glioma patients by using publicly available tumour databases. Additionally, we examined the impact on immune infiltrating cells, immune checkpoints and chemokines within the glioma. The findings indicate the potential involvement of disulfidptosis in glioma and offer a novel approach for personalised and accurate treatment of glioma.

## 2 Methods and Materials

### 2.1 Data Source

According to a recent report, we selected GYS1, LRPPRC, NCKAP1, NDUFA11, NDUFS1, NUBPL, OXSM, RPN1, SLC3A2 and SLC7A11 to constitute the disulfidptosis gene set [14].

Glioma data were obtained from multiple public databases, including TCGA (The Cancer Genome Atlas, https://portal.gdc.cancer.gov/), CGGA (The Chinese Glioma Genome Atlas, http://www.cgga.org.cn/) and the Rembrandt database (https://www.cgga.org.cn/download_other.jsp). Additionally, transcriptome data from normal patients were retrieved from the GTEx database (https://xenabrowser.net/datapages/) and the CGGA database. TCGA data were primarily used for model training, while the CGGA and Rembrandt databases served as external validation cohorts. A total of 1031 GBM samples and 1172 normal samples are enrolled in this study.

### 2.2 Expression Analysis and Survival Analysis

In the training set, we compared the expression of 10 disulfidptosis genes between normal brain tissue and glioma. Subsequently, an analysis of the effects of 10 disulfidptosis genes on overall survival (OS) was conducted using the Kaplan–Meier survival curve. Moreover, we employed the external validation set CGGA database to verify our results.

### 2.3 Establishment of Risk Score

A univariate Cox regression was conducted to analyse the influence of 10 disulfidptosis genes on patients' OS. Subsequently, Lasso regression was utilised to identify the optimal gene combination, with the genes having nonzero β values being further selected by multivariate Cox regression. For each patient in the training and validation sets, the risk score can be determined by utilising the regression coefficients and mRNA expression values of each gene. The formula for this calculation is as follows: Risk score = (βmRNA1 × expmRNA1 + βmRNA2*expmRNA2 + … + βmRNAn × expmRNAn). Upon determining the median risk score, patients were divided into two groups: a high‐risk score group and a low‐risk score group, and further research was conducted.

### 2.4 Evaluation, Validation and Clinical Significance of the Risk Score

The impact of the risk score on overall survival (OS) was assessed using Kaplan–Meier survival curves and time‐dependent ROC analysis for 1‐, 3‐ and 5‐year OS. A nomogram was constructed based on Cox regression analysis, with its predictive accuracy evaluated via calibration curves and decision curve analysis (DCA). Additionally, the risk score's correlation with clinicopathological features was examined, and its validity was confirmed using an external dataset.

### 2.5 GSEA Enrichment Analysis

Grouping the patients in two external validation sets according to their high‐ and low‐risk scores, the ‘limma’ package was used to analyse the differentially expressed genes (DEGs). To enhance the accuracy of the DEG enrichment analysis, the intersection of the DEGs from both the external validation sets was conducted for GO function, KEGG pathway and GSEA enrichment analysis. Representing the top 10 results, bubble charts and bar charts were presented.

### 2.6 Examining the Link Between Risk Score and Tumour Immunity

We analysed immune cell infiltration across risk groups using Cibersort, ssGSEA and Estimate, and assessed correlations between immune checkpoints, chemokines and risk scores. TIDE was used to evaluate immune therapy response between groups. We also examined 10 disulfidptosis genes across cell types using TISCH and analysed their association with immune infiltration in gliomas using TIMER.

### 2.7 Identified the Hub Gene of Model

To identify the hub gene of the model for future analysis and to explore the potential regulatory mechanisms of GBM, we conducted a comprehensive analysis. This included a literature review and an evaluation of the coefficients from the integrative model. As a result, RPN1 was confirmed as the key candidate gene for further investigation.

### 2.8 Cell Culture

Glioblastoma cell lines U87, U251, U118, U138 and normal astrocytes HA1800 were derived from Zhongqiao Xinzhou Biological Co., LTD. (Shanghai). Complete culture medium was used for cell culture and passage. The cells were routinely placed in a 37°C incubator containing 5% CO_2_.

### 2.9 Plasmid Construction

The RPN1RNA interference plasmid was constructed by Genechem Co. Ltd. (Shanghai). According to the instructions, the plasmid was transfected into U87 and U251 cells with lipo3000. After 48 h, the cells were collected to verify the knockdown effect.

### 2.10 Western Blotting

The equivalent amount of cell protein was taken for SDS‐page electrophoresis. After electrophoresis, membrane transfer and sealing, the protein samples were incubated with RPN1 rabbit primary antibody (1:1000, Proteintech) and antirabbit secondary antibody (1:2000 abcam). We used beta‐actin (1:2000, abcam) as the internal reference. The experimental results were analysed using ImageJ software. In addition, we observed the protein expression levels of RPN1 in the CPTAC database (https://ualcan.path.uab.edu/analysis‐prot.htm).

### 2.11 MTT, Clony Formation Assay, Wound Healing Assay and Transwell Assay

U87 and U251 cells were divided into the shNC group and the shRPN1 group, and cytological experiments were performed 48 h after transfection. To put it simply, 2 × 10^3^ cells were planted in each hole of the 96‐well plate. After the cells were attached to the wall, MTT reagent and DMSO were added successively to measure the absorbance value and take this value as the starting value. The absorbance values of 1, 2 and 3 days of the two groups of cells were measured by the same steps.

5 × 10^2^ cells were implanted in six‐well plates with three pores in each group. The complete medium was replaced every 3 days. After 12 days, following fixation with paraformaldehyde, the cells were stained with crystal violet to count the colonies in the two groups.

5 × 10^5^ cells were implanted in a six‐well plate. Scratches were made using a 200‐μl pipette tip. The scratch width measured under the microscope was used as the initial value. The scratch width was measured again 24 h after the culture was continued using base medium.

A single‐cell suspension of 2 × 10^4^ cells was prepared using basic medium and placed in the upper chamber of the Transwell chamber. Then the chamber was placed in a 24‐well plate containing a complete medium and cultured for 48 h. The number of cells in six regions of each Transwell chamber in both groups was observed under a microscope.

### 2.12 Statistical Analysis

Statistical analyses were performed using R (4.2.1). Risk scores were computed via univariate Cox, LASSO and multivariate Cox regression. Pearson's correlation, chi‐square test, t‐test and ANOVA were used for comparisons. Kaplan–Meier curves were analysed with the log‐rank test. *p* < 0.05 was considered significant.

## 3 Result

### 3.1 The Expression of Disulfidptosis Genes Was Significantly Changed in Glioma

**FIGURE 1:**
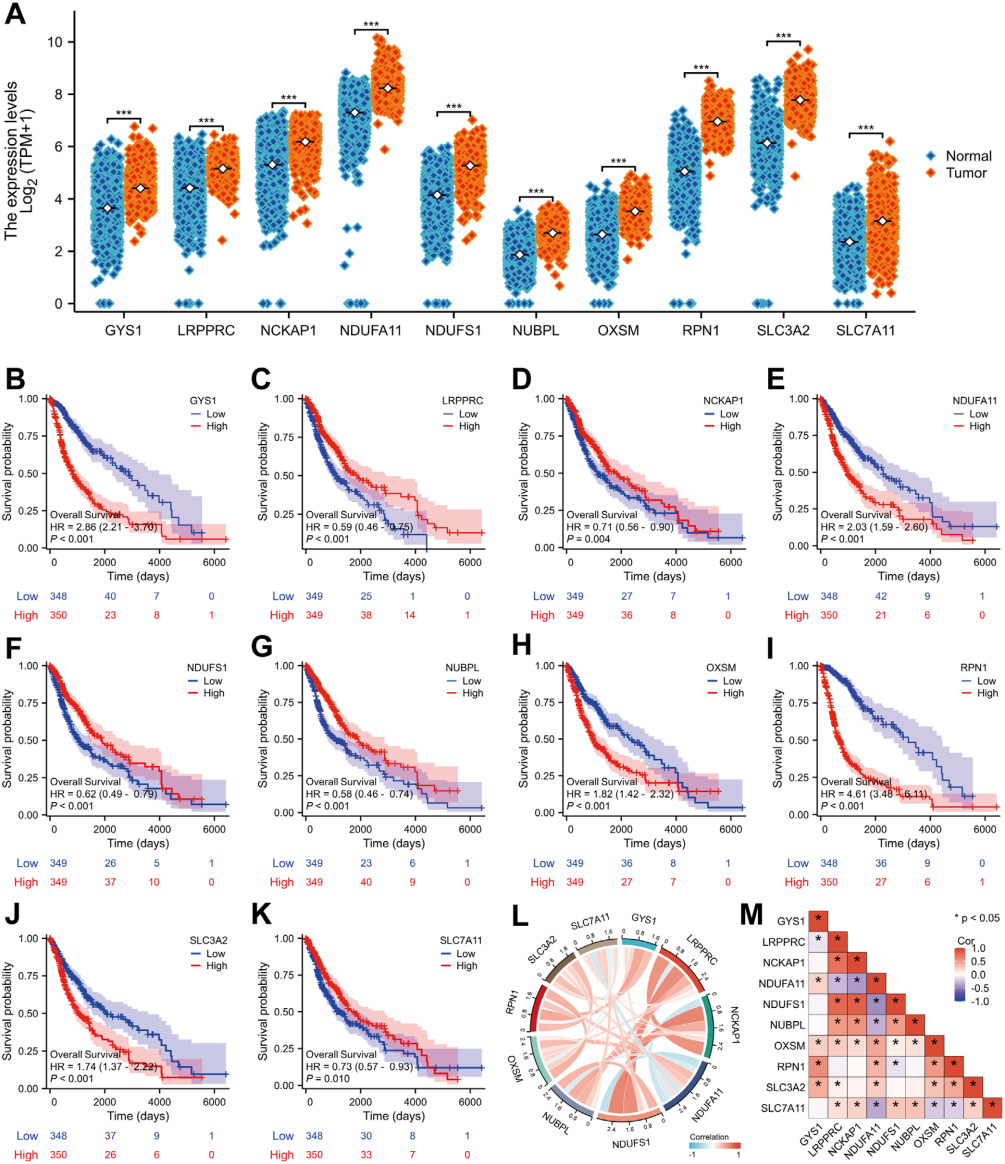
Expression and survival analysis of disulfide death gene in glioma. (A) mRNA expression levels of 10 disulfidptosis genes in the TCGA database were significantly upregulated in glioma. (B–K) Kaplan–Meier survival analysis of 10 disulfidptosis genes in glioma. (L–M) Correlation analysis of 10 disulfidptosis genes. TCGA, The Cancer Genome Atlas. *p < 0.05, ***p < 0.001.

Figure S1 shows our analysis flow. In the TCGA database, mRNA expression levels of GYS1, LRPPRC, NCKAP1, NDUFA11, NDUFS1, NUBPL, OXSM, RPN1, SLC3A2 and SLC7A11 were all significantly increased (Figure 1A). Survival analysis revealed that patients with high expression of GYS1, NDUFA11, OXSM, RPN1 and SLC3A2 had significantly shorter OS, while those with high expression of LRPPRC, NCKAP1, NDUFS1, NUBPL and SLC7A11 had longer OS (Figure 1B–K). It is proposed that these genes could be essential for the advancement of glioma. Correlation analysis of 10 disulfidptosis genes showed a significant negative correlation between OXSM and SLC7A11, and a significant positive correlation between OXSM and other genes. NDUFA11 and LRPPRC, NCKAP1, NDUFS1, NUBPL and SLC7A11 had a significant negative correlation relationship, and GYS1, OXSM, RPN1 and SLC2A2 had significant positive correlation. It was suggested that OXSM and NDUFA11 may be the key genes in this gene cluster (Figure 1L,M).

Survival analysis of the external validation set (CGGA) showed that the prognostic value of 10 disulfidptosis genes for glioma patients' OS was consistent with that of the training set (Figure S2A–J). Additionally, the mRNA levels of 10 disulfidptosis genes varied with WHO grade, with GSY1, NDUFA11, OXSM and RPN1 increasing and SLC7A11 decreasing with higher WHO classification (Figure S2K,L). In patients with IDH mutation, GSY1, NDUFA11 and OXSM expression levels were lower, while LRPPRC, NCKAP1, NDUFS1 and NUBPL were higher (Figure S2M,N). Furthermore, GSY1, NDUFA11 and RPN1 expression levels decreased in 1p/19q codeletion patients, while LRPPRC, NDUFS1 and SLC3A2d increased (Figure S2O,P).

### 3.2 Establishment of Risk Score Associated With Disulfidptosis Genes

**FIGURE 2:**
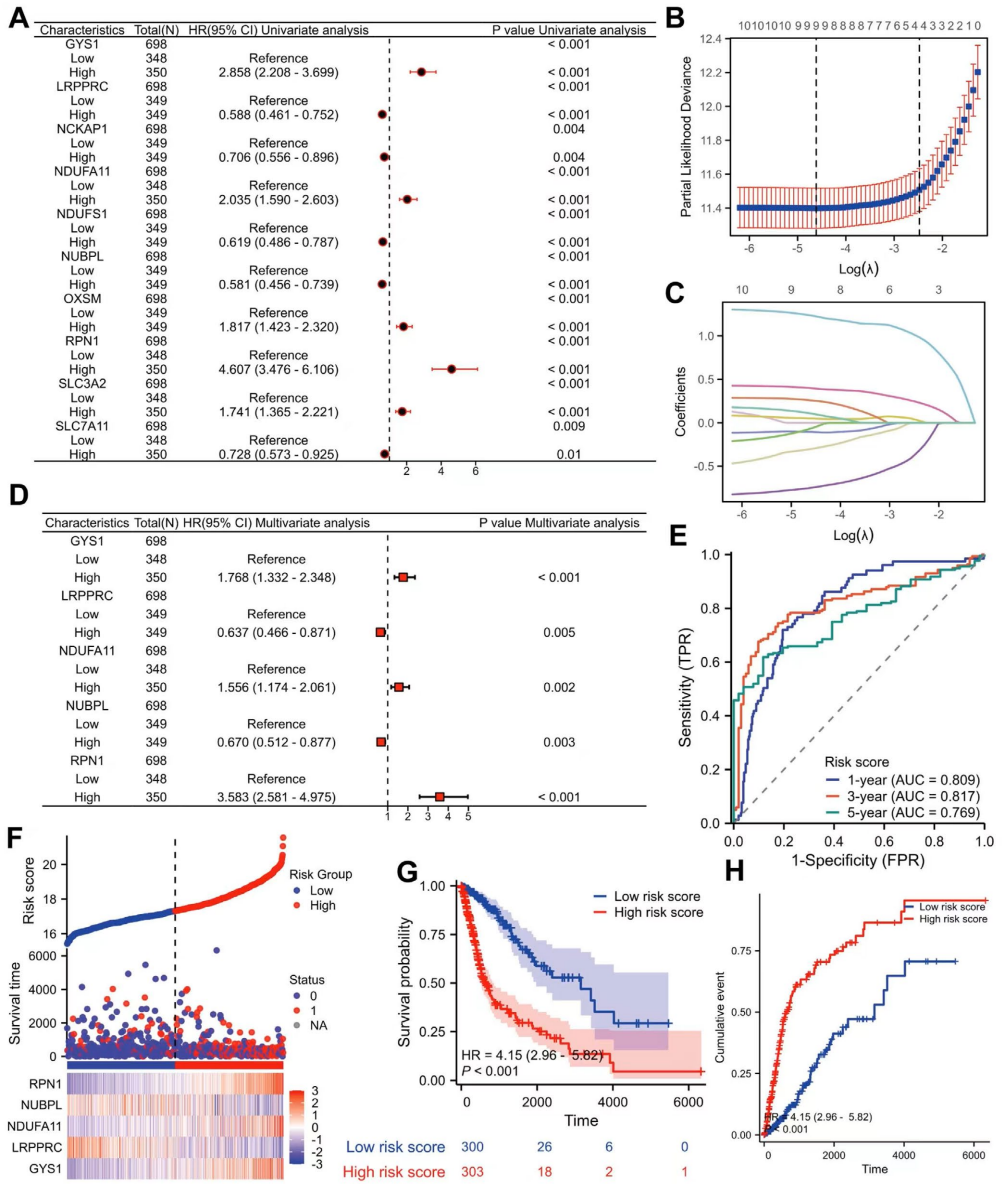
Establishment of disulfidptosis genes‐associated risk scores. (A) Preliminary screening of 10 disulfidptosis genes by Univariate Cox regression analysis. (B) Selection plot of Lasso regression coefficients. (C) Plot of the trajectories of the Lasso regressors. (D) Multivariable Cox regression analysis to determine the established disulfidptosis genes‐associated risk score. (E) Time ROC analysis showed that the risk of glioma patients 1‐, 3‐ and 5‐year OS has good predictive value. (F) Risk factor map shows differences between high‐ and low‐risk score groups of patients' survival condition. RPN1, NDUFA11 and GYS1 show high expression in the high‐risk group, and NUBPL and LRPPRC show high expression in the low‐risk group (0: Alive, 1: Dead). (G) Kaplan–Meier survival analysis showed that patients with high‐risk scores have short lifetimes. (H) Kaplan–Meier survival analysis shows cumulative events of high‐risk score patients who have died. ROC, receiver operating characteristic.

The survival of glioma patients was significantly influenced by all 10 genes associated with disulfidptosis according to the results of the univariate Cox regression analysis (Figure 2A). Further screening by Lasso regression analysis identified nine genes (Figure 2B,C), and then multivariable Cox regression analysis was employed to select five genes (GSY1, LRPPRC, NDUFA11, NUBPL and RPN1) to create a risk score (Figure 2D). Based on the median risk score, the training and validation sets were categorised into groups of low or high risk. Time‐dependent ROC analysis showed that the AUC for OS in the training set was 0.809, 0.817 and 0.769 for 1, 3 and 5 years respectively (Figure 2E). The risk factor maps showed that the high‐risk score group had more deaths. GSY1, NDUFA11 and RPN1 were expressed at a high level in the high‐risk score group, while LRPPRC and NUBPL were expressed at a low level (Figure 2F). The Kaplan–Meier analysis revealed that individuals belonging to the high‐risk score category experienced noticeably reduced OS and a higher cumulative event of death (Figure 2G,H).

In the external validation sets (CGGA and Rembrandt), the risk factor maps revealed that the high‐risk score group had a higher mortality rate and the gene expression distribution was the same as the training set (Figure S3A,D). The Kaplan–Meier survival curve also showed similar results as the training set (Figure S3B,E). According to time‐dependent ROC analysis, the AUC of OS in glioma patients predicted by risk score was 0.763, 0.822, 0.824 and 0.702, 0.772, 0.778 respectively (Figure S3C,F). The risk score proved to be more effective than the prediction performance of five genes alone for glioma 1‐, 3‐ and 5‐year OS in both the training and validation sets (Figure S3G–I).

### 3.3 Nomogram Construction and Verification to Include Risk Score

We constructed a Nomogram that included WHO grade, 1p/19q codeletion states and risk score to assess its accuracy through calibration curve (Figure S4A,C). The Nomogram achieved a C‐index of 0.784 in the TCGA database (Figure S4B), while in the CGGA database, it achieved a C‐index of 0.757 (Figure S4D), demonstrating the model's high accuracy. In the TCGA database, the DCA revealed that the Nomogram had significantly higher net benefits for predicting glioma patients' outcomes than other clinical variables (Figure S4E–G).

### 3.4 The Application Value of Risk Score in Clinical Characteristics

In subgroup survival analysis of gender, age and 1p/19q, patients in the high‐risk score group all had shortened OS (Figure S5A–F). In WHO II‐III patients, patients in the high‐risk score group also had shortened OS (Figure S5G). Moreover, risk score increased with the increase of WHO degree of glioma (Figure S5H). A marked distinction was observed in the risk scores of IDH mutation, 1p/19q codeletion, MGMT methylation and age (Figure S5I–L), whereas risk score did not have a significant difference in gender (Figure S5M).

### 3.5 Enrichment Analysis of DEGs

**FIGURE 3:**
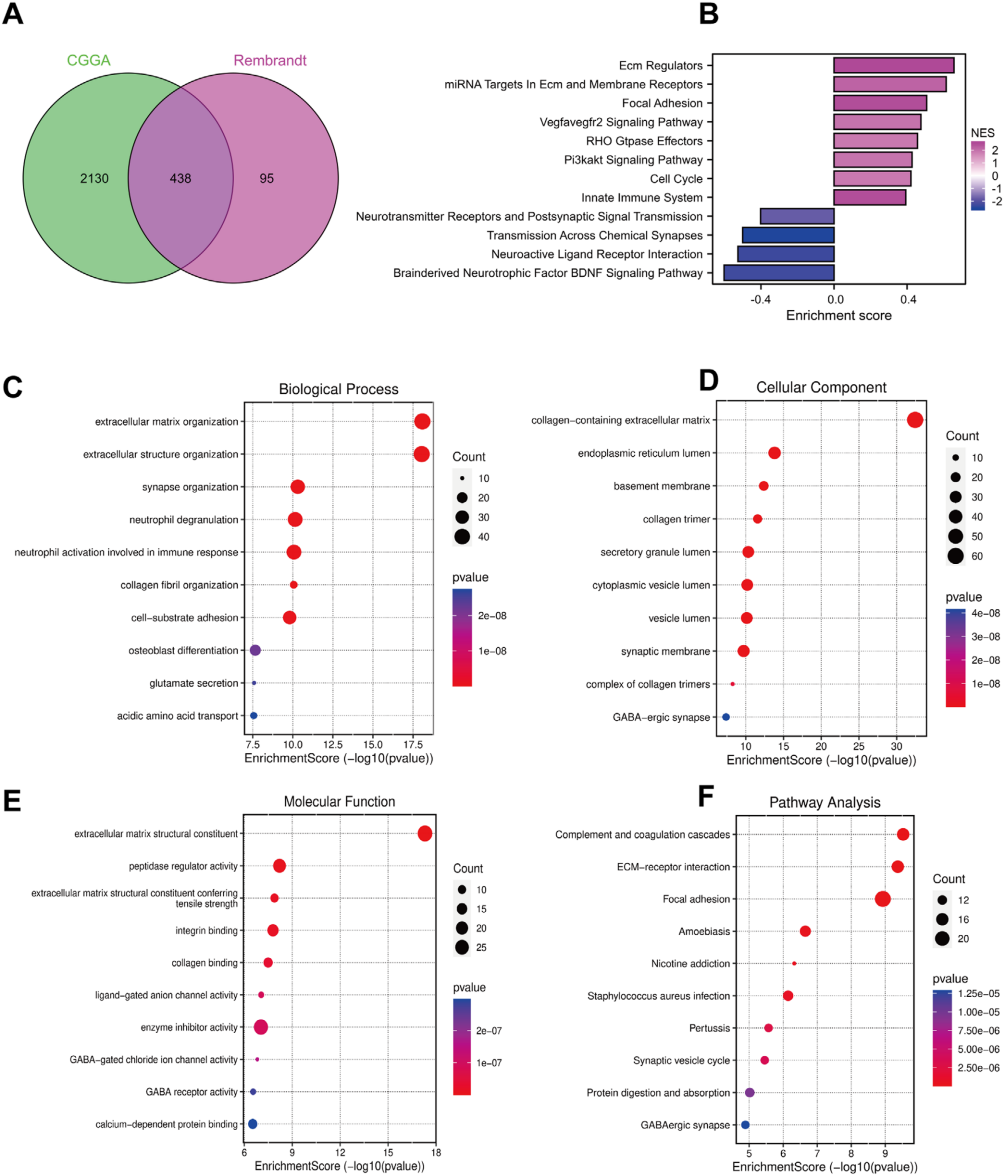
Enrichment analysis of differentially expressed genes between high–low‐risk groups. (A) The Venn diagram shows the intersection of differentially expressed genes in the TCGA and Rembrandt databases. (B) GSEA enrichment analysis. (C–E) GO function enrichment analysis. (F) KEGG pathway enrichment analysis. BP, biological process; CC, cellular components; GSEA, gene set enrichment analysis; KEGG, kyoto encyclopedia of genes and genomes; MF, molecular function; TCGA, The Cancer Genome Atlas.

In two external validation sets, a total of 438 DEGs were identified (Figure 3A). The findings of GSEA indicated that the ECM regulation, miRNA targets in ECM, membrane receptor, focal adhesion, VEGF2 signalling pathway, Rho gtpase effectors, PI3K/AKT signalling pathway, cell cycle and innate immune system were all linked to the high‐risk score group (Figure 3B). Functional analysis using GO revealed significant enrichment of biological processes (BP) including organisation of the extracellular matrix, organisation of extracellular structures, organisation of synapses, degranulation of neutrophils, activation of neutrophils involved in immune response and adhesion to cell substrates (Figure 3C). Significantly enriched in cellular components (CC) were extracellular matrix containing collagen, lumen of endoplasmic reticulum, secretory granule, cytoplasmic vesicle and synaptic membrane (Figure 3D). Additionally, extracellular matrix structural constituent, peptidase regulator activity, integrin binding and enzyme inhibitor activity were significantly enriched in molecular function (MF) (Figure 3E). KEGG pathway enrichment analysis showed that complement and coagulation cascades, ECM receptor interaction, focal adhesion and amoebiasis were significantly enriched (Figure 3F).

### 3.6 Influence of Risk Score on Tumour Immune Microenvironment

**FIGURE 4:**
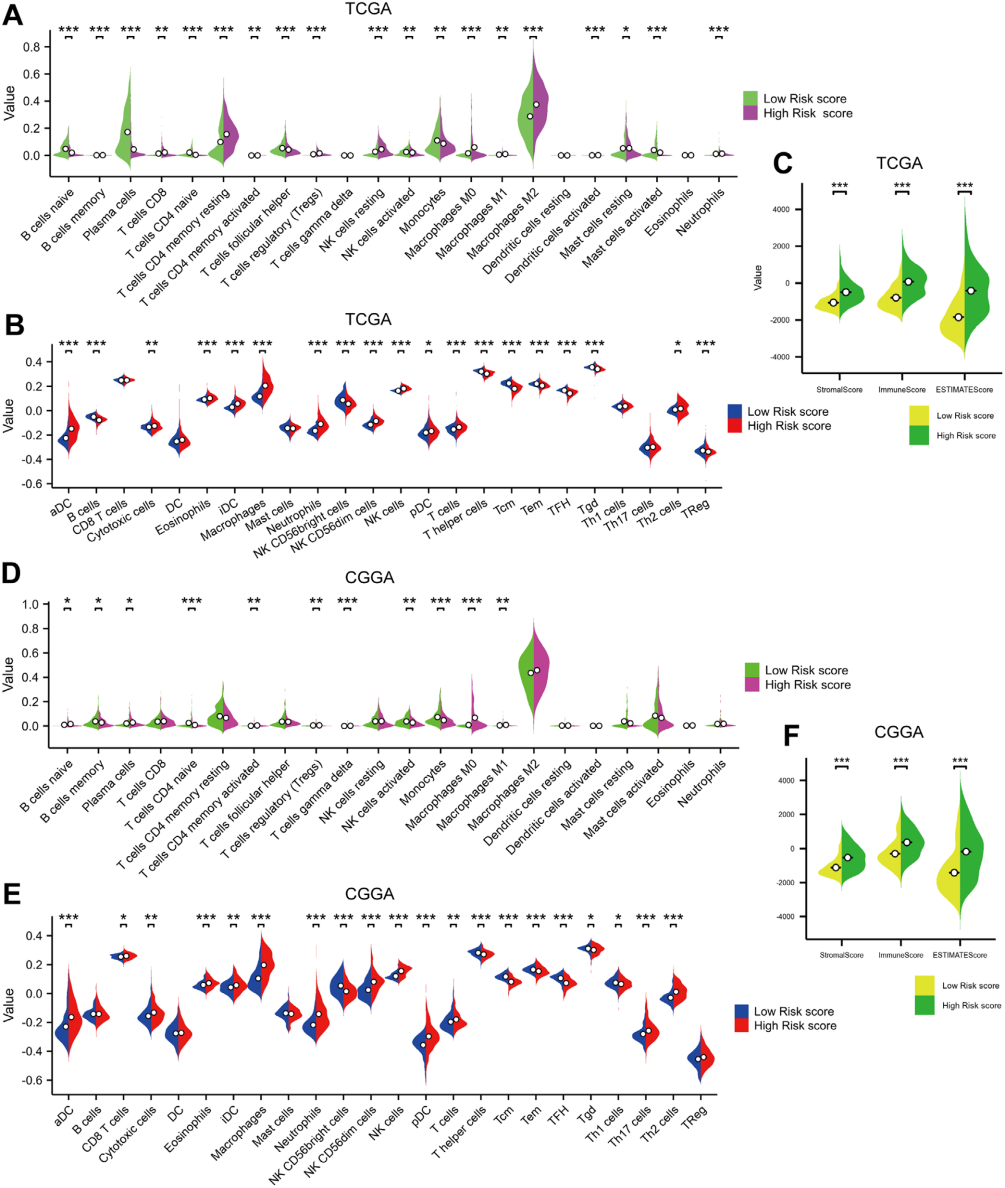
Relationship between risk score and tumour immune microenvironment. (A) In the TCGA database, the Cibersort algorithm showed significant differences in risk scores across 20 types of immunoinfiltrating cells. (B) The ssGSEA algorithm showed significant differences in risk scores among 19 types of immunoinfiltrating cells. (C) The estimate algorithm showed that the matrix scores, immune scores and estimated scores of the high‐risk group were higher than those of the low‐risk group. (D) In the CGGA database, the Cibersort algorithm showed significant differences in risk scores among 11 types of immunoinfiltrating cells. (E) The ssGSEA algorithm showed significant differences in risk scores across 20 types of immunoinfiltrating cells. (F) The Estimate algorithm also showed that patients in the high‐risk rating group had higher matrix scores, immune scores and estimated scores. CGGA, The Chinese Glioma Genome Atlas; TCGA, The Cancer Genome Atlas. *p < 0.05, **p < 0.01, ***p < 0.001.

Results from the TCGA and CGGA databases, when evaluated with the Cibersort, ssGSEA and Estimate algorithms, showed marked disparities in immune infiltrating cells between different risk score groups. According to the Cibersort algorithm, there were significant variations in the presence of immune infiltrating cells between the high‐ and low‐score groups in the TCGA database, with a total of 20 different types. In contrast, the CGGA database showed 11 distinct types (Figure 4A,D). The ssGSEA algorithm revealed that there were 19 and 20 types of immune infiltrating cells with significant differences between the high and low score groups in the TCGA and CGGA databases respectively (Figure 4B,E). The estimate method showed that in both datasets, the high‐risk score group exceeded the low‐risk score group in terms of matrix score, immunological score and estimated score (Figure 4C,F).

### 3.7 Correlation of Risk Score With Immune Checkpoints and Chemokines, and Immune Therapy Response

**FIGURE 5:**
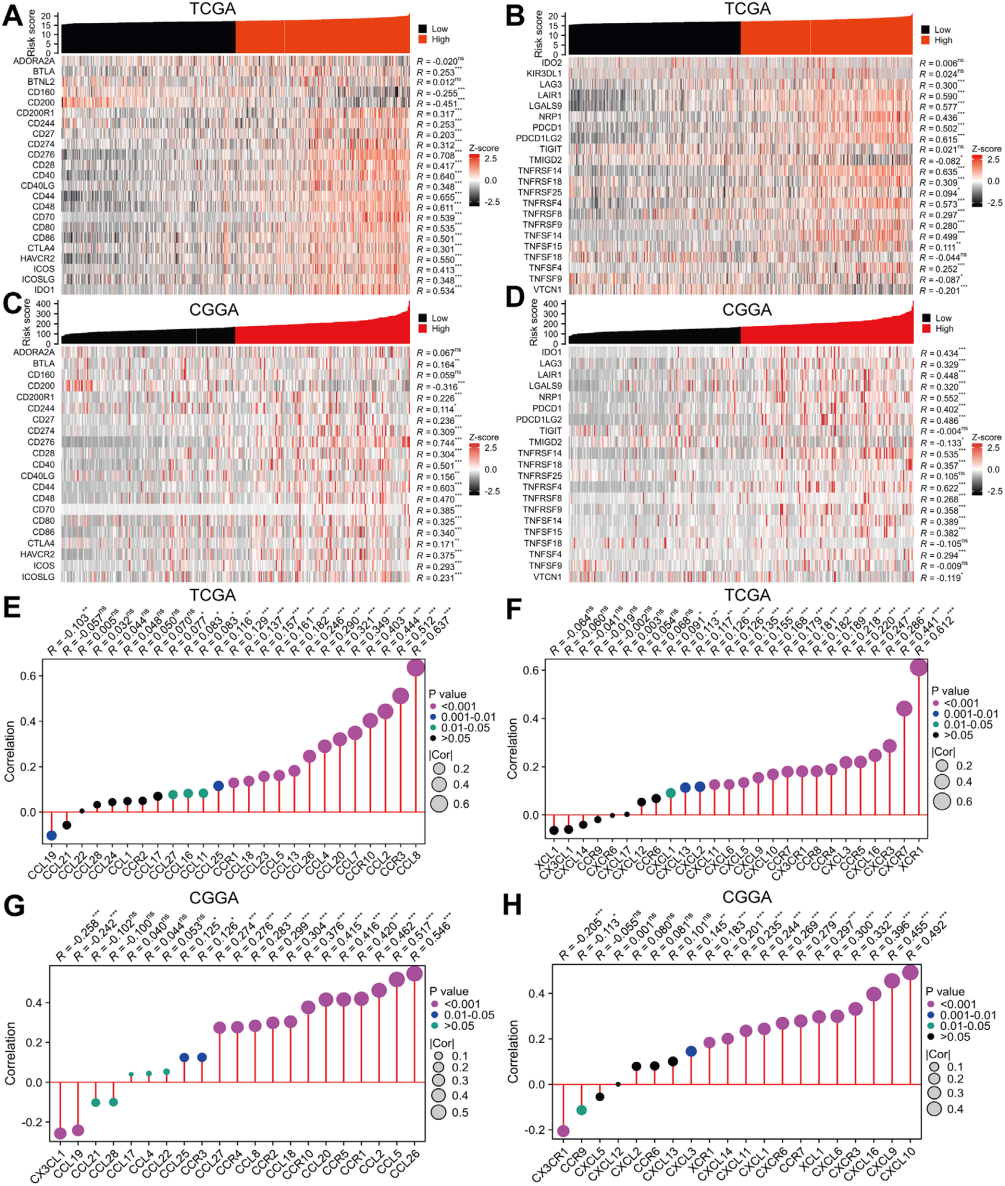
Risk scores can characterise immune checkpoint and chemokine expression levels in gliomas. (A and B) Coexpression heat maps of risk scores and 46 common immune checkpoints in the TCGA database. (C and D) Coexpression heat maps of risk scores and 42 common immune checkpoints in the CGGA database. (E and F) Lollipop charts of correlation between risk scores and 51 common chemokines in the TCGA database. (G and H) Lollipop chart of correlation between risk scores and 41 common chemokines in the CCGA database. CGGA, The Chinese Glioma Genome Atlas; ns, no signification; TCGA, The Cancer Genome Atlas. *p < 0.05, **p < 0.01, ***p < 0.001.

Analysis of the TCGA and CGGA databases revealed a robust association between risk score and 45 common immune checkpoints in the glioma immunosuppressive microenvironment, as demonstrated by the coexpression heat maps (Figure 5A–D). Risk score was significantly positively correlated with CD274, CD276, CTLA4, LAG3, LGALS9, PDCD1, PDCD1LG2 and TNFRSF4 in both databases. Additionally, the TCGA database indicated a notable and affirmative association between risk score and CCL8, CCL7, CCL5, CCL2, CCR3, XCR1, CXCR7 and so on (Figure 5E,F) while the CGGA database demonstrated a notable association between risk score and CCL26, CCL20, CCL5, CCL2, CCR1, CCR5, CCR10, CXCL10, CXCL9, CXCL16 and so on (Figure 5G,H). In addition, our results also demonstrate that high‐risk score could benefit from immune therapy, with a low TIDE score (Figure S6).

### 3.8 Relationship Between Disulfidptosis Genes and Tumour Microenvironment

**FIGURE 6:**
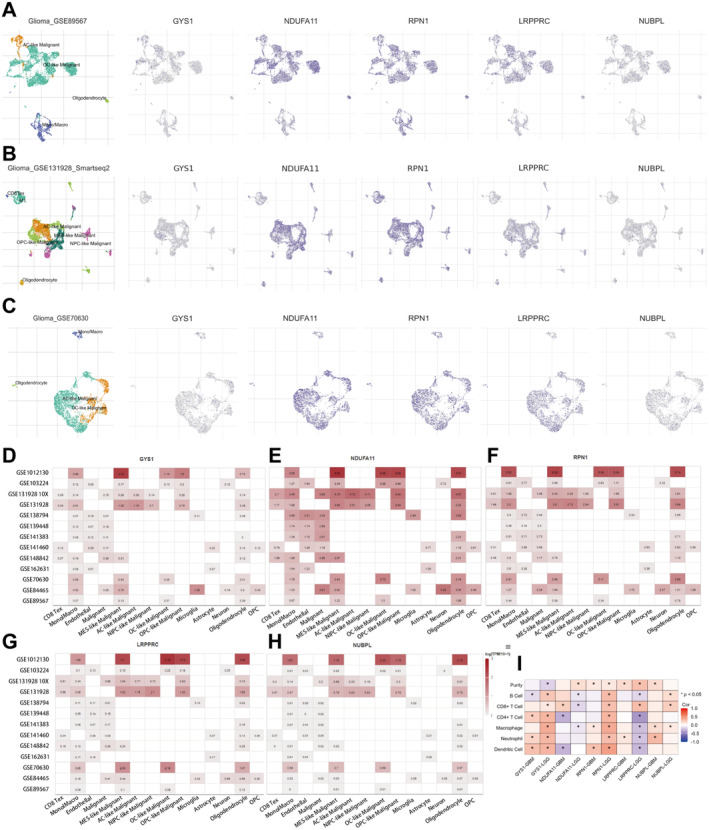
Relationship between five disulfidptosis genes and tumour immune infiltrating cells. (A–C) In TISCH database, the expression and distribution of five disulfidptosis genes in GSE89567, GSE131928 Smartseq2 and GSE70630. (D–H) In TISCH database, five disulfidptosis genes were expressed in 14 cell types in 13 single‐cell sequencing data sets. (I) Analysis of the correlation between five disulfidptosis genes and tumour purity and degree of immune cell infiltration in low‐grade gliomas and glioblastomas in TIMER database.

TISCH enables the exploration of TME across different cancer types. Our results show that these genes were mainly expressed in mono/macro, malignant and oligodendrocyte (Figure 6A–H). Furthermore, the TIMER database revealed a correlation between tumour purity and immune cell infiltration (including B cell, CD8+ T cell, CD4+ T cell, macrophage, neutrophil, dendritic cell) in GBM and low‐grade glioma, with five disulfidptosis genes involved (Figure 6I).

### 3.9 Preliminary Verification of Genes Related to Disulfidptosis

**FIGURE 7:**
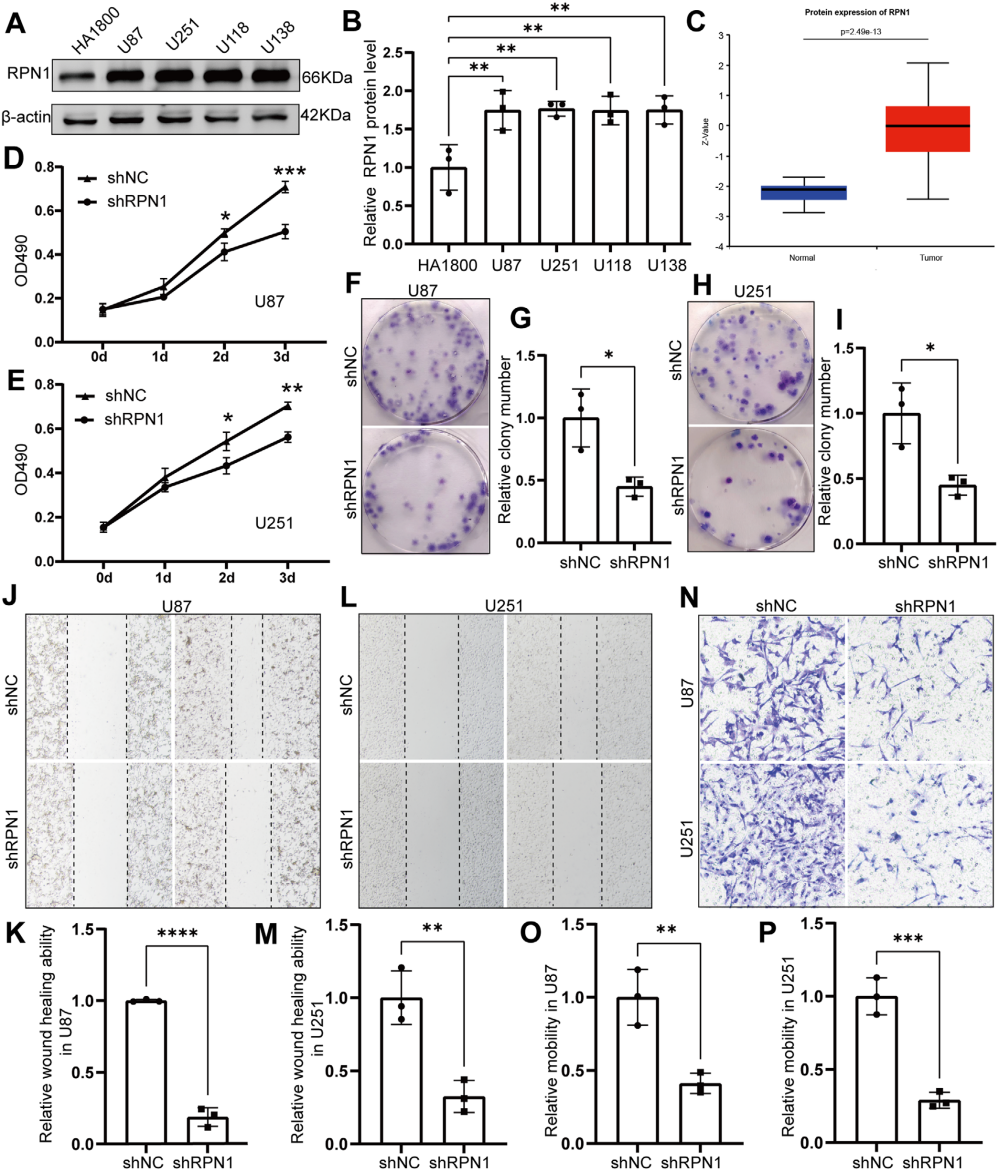
RPN1 significantly promoted the proliferation and migration of glioma cells. (A–C) The protein levels of RPN1 in U87, U251, U118 and U138 were significantly upregulated, which was consistent with the results in the CPTAC database. (D and E) The proliferation ability of U87 and U251 cells decreased after the RPN1 protein level was knocked down. (F–I) When RPN1 protein levels were knocked down, colony formation in U87 and U251 cells was reduced. (J–M) After knocking down RPN1 protein levels, the wound healing ability of U87 and U251 cells was reduced. (N–P) The migration ability of U87 and U251 cells was reduced after the RPN1 protein level was knocked down. *p < 0.05, **p < 0.01, ***p < 0.001, ****p < 0.0001.

In our study, the protein expression level of RPN1 was significantly increased in glioblastoma cell lines U87, U251, U118 and U138 (Figure 7A,B). This was consistent with what we observed in the CPTAC samples (Figure 7C). After the RPN1 protein level was knocked down by shRNA, we found that the proliferation, clonal formation and cell migration of U87 and U251 cells were significantly decreased (Figure 7D–P).

## 4 Discussion

Compared to traditional surgery, radiotherapy and chemotherapy, immunotherapy has emerged as a crucial treatment option for gliomas [15, 16]. Nevertheless, the tumour immunosuppressive microenvironment in which gliomas are located significantly reduces the effectiveness of the therapy [17]. Therefore, searching for biomarkers and targets that can enhance the efficiency of glioma therapy has great potential for clinical application. Numerous studies have been conducted to manage PCD in glioma to promote tumour cell death, including ferroptosis, pyroptosis and autophagy [18, 19, 20, 21, 22]. Recently, disulfidptosis has been recognised as a new type of cellular death, providing a potential avenue for targeted therapies of tumours [3]. Nevertheless, the unclear prognostic and clinical consequences of disulfidptosis genes in gliomas remain uncertain. To tackle this issue, we created a risk score using a publicly available dataset, enabling the prediction of glioma patients' prognosis. In addition, our findings demonstrated a correlation between the risk score and the level of immune cell infiltration into the tumour, along with the expression of immune checkpoints and chemokines. The results of our study indicate that the established risk score is highly predictive, and genes associated with disulfidptosis may serve as promising biomarkers and therapeutic targets for gliomas.

In fact, cystine/glutamate reverse transporters System Xc‐ and SLC7A11 have been intensively studied in gliomas [23]. SLC7A11 encodes a transmembrane protein that facilitates the reverse transport of cystine and glutamate, which helps to prevent ferroptosis by increasing the production of GSH. This protein has been found to be overexpressed in a variety of human cancers, making it a potential biomarker for cancer immune infiltration and poor prognosis [24, 25, 26]. Glutamate released by System Xc has been found to play a role in the infiltration of GBM cells, and the increased cystine uptake by glioma cells has been linked to enhanced antioxidant capacity, which can protect tumour cells from the oxidative damage caused by radiotherapy and chemotherapy, thus promoting tumour invasion and recurrence [27, 28]. In gliomas, System Xc‐ is generally expressed at higher levels, and the rate of cystine uptake is faster than the release rate of glutamate, yet the expression of the glutamate transporter is downregulated. This leads to an accumulation of extracellular glutamate, causing excitotoxic cell death in the surrounding nontumour tissues [29]. Blocking the uptake of cystine causes a decrease in the levels of GSH, resulting in an increase in ROS, DNA double bond breaking and cell death, thereby boosting the effectiveness of radiation therapy [30]. Furthermore, blocking System Xc‐ has been shown to increase the efficacy of temozolomide and reduce glioma cell proliferation and survival [31]. Our study demonstrated a marked elevation in the mRNA levels of 10 disulfidptosis genes in glioma. Results from the survival analysis indicated that shorter survival periods were associated with high expression levels of GYS1, NDUFA11, OXSM, RPN1 and SLC3A2, while increased survival periods were linked to high expression levels of LRPPRC, NCKAP1, NDUFS1, NUBPL and SLC7A11. We validated our findings in an external validation set, where we obtained results that were in line with the training set. GYS1, NDUFA11, OXSM, RPN1, and SLC3A2 play a role in promoting cancer in glioma, while LRPPRC, NCKAP1, NDUFS1, NUBPL and SLC7A11 play a role in inhibiting tumour. We also revealed that the 10 genes were closely positively or negatively correlated with each other, and there were notable differences between different clinical features, indicating that the disulfidptosis genes included in this study have significant clinical application value, which provides a basis for our further research.

SLC7A11, the core gene involved in disulfidptosis, was found to be an independent risk predictor of GBM [27, 32, 33, 34]. Studies have shown that the expression of SLC7A11 in gliomas can contribute to tumorigenesis, progression and resistance to conventional chemotherapies [35]. Lowering of SLC7A11 expression had been linked to increased ROS levels, resulting in greater cell death due to oxidative and genotoxic stress, and enhanced invasive properties. However, overexpression of SLC7A11 had also been linked to increased resistance to oxidative stress, reduced sensitivity to temozolomide, decreased endogenous ROS levels, reduced migration and invasion, changes in the actin cytoskeleton and increased cancer stem cell‐like characteristics [31, 35, 36]. Moreover, high expression of SLC7A11 caused significant peritumoral glutamate excitotoxicity, resulting in glioma‐associated seizures and shorter overall survival. It was also an independent indicator of seizures at glioma diagnosis [31, 37, 38, 39]. In our study, the high expression of SLC7A11 suggests that patients had longer OS, suggesting that disulfidptosis involved in SLC7A11 may play an important role in glioma, which also provided a theoretical hypothesis for further study of disulfidptosis in glioma.

The risk score linked to disulfidptosis was assessed and confirmed in both the training set and validation set. According to our findings, the risk score emerged as an independent risk factor contributing to the unfavourable prognosis of individuals diagnosed with glioma. The survival analysis indicated that the OS of the group with a high‐risk score was comparatively shorter. Time‐dependent ROC analysis demonstrated that the risk score exhibited excellent predictive efficacy for OS at 1, 3 and 5‐year intervals. The precision of the risk score exceeded that of an individual gene. The risk factor maps also indicated that the majority of deceased patients were concentrated within the high‐risk score group. The levels of GYS1, NDUFA11 and RPN1 were notably increased in the high‐risk score group, while NUBPL and LRPPRC were seen to be elevated in the low‐risk score group. This finding is in alignment with the survival analysis, implying that NUBPL and LRPPRC may be cancer suppressors in gliomas. The nomogram model of risk score also demonstrated good accuracy and clinical net benefits for the prediction of OS. The risk score also had significant differences in different clinical features. In conclusion, the risk score can be used as an ideal indicator for the OS of glioma patients. Of the five gene signatures we included, RPN1 is a key element of the OST complex, playing a vital role in N‐linked glycosylation. In vitro, the inhibition of RPN1 results in decreased proliferation and invasion of breast cancer cells, along with the induction of apoptosis caused by endoplasmic reticulum stress [40]. However, the specific role of gene signature in glioma, including RPN1, has yet to be determined. In our study, we found that the expression of RPN1 increased in glioma cells and had significant effects on the proliferation and migration of glioma cells. Further experiments should be conducted to explore how these genes affect the malignant biological characteristics of gliomas by influencing the disulfidptosis process, providing definitive evidence for disulfidptosis as a treatment for gliomas.

The interaction between glioma cells and the extracellular matrix (ECM) is essential for glioma cell invasion [41, 42]. Glioma cells use particular adhesion receptors to attach to and move along the components of the brain ECM [43]. ECM regulatory mechanisms can also lead to immune rejection, making immunotherapy difficult to carry out [41]. Cell migration is heavily reliant on focal adhesion complexes, which act as the nucleus of several signalling pathways [44]. Research has shown that neutrophils, which are an integral part of the tumour microenvironment, are closely associated with the development of cancerous tumours and the immunosuppression of gliomas [45, 46]. Glioma‐neutrophil communication can sustain long‐term tumour activation [47]. Our enrichment analysis on the high‐risk score group evidenced a notable increase in ECM, focal adhesion and neutrophil activation, thus confirming the accuracy of the risk score we formulated to identify the malignant characteristics of gliomas.

Tumour immune microenvironment is one of the important characteristics affecting tumour occurrence, invasion, metastasis and drug resistance [48]. Gliomas are infiltrated by immune cells consisting of microglia (resident cells of the central nervous system) as well as peripheral macrophages, granulocytes, bone marrow–derived suppressor cells and T lymphocytes [49]. To better understand the differences in tumour immunoinfiltrating cells between risk scores, we used a variety of algorithms to evaluate the TME. The expression of T helper cells, active Tcm, Tem, TFH, Tgd, NK cells and monocytes decreased in the high‐risk score group in both the training and validation sets. Conversely, the expression levels of eosinophils, iDC, macrophages, neutrophils, NK CD56dim cells, NK cells, pDC and T cells were all increased. The intratumoral density of glioma‐associated microglia/macrophages (GAM) and MDSC is the highest in gliomas and is inversely correlated with patient survival [49]. Our findings could, to a certain extent, explain why the high‐risk score group has a shorter OS. Moreover, the high‐risk group exhibited elevated matrix, immune and estimated scores, signifying that the risk score can precisely reflect the degree of immune cell infiltration in patients.

The severe immunosuppression of glioma in the local and systemic scope of the tumour is one of the important reasons affecting the effect of immunotherapy [50]. A key factor in this is the expression level of immune checkpoint molecules [51, 52]. The immune system's evasion by tumour cells could potentially explain the lack of successful treatment for gliomas [49]. Chemokines, expressed by stromal cells or produced by glioma cells, may influence tumour cell migration, invasion, proliferation, angiogenesis and immune cell infiltration [53]. Chemokines, in particular, can regulate immune cell transport by shaping local networks [54].

## 5 Conclusion

Our model has good predictive value and clinical application potential for glioma patients' survival and can help improve the accuracy of treatment decisions.

## Author Contributions

The study was conceived and designed by X.N., W.S., L.H. and Z.Y., and they also drafted the manuscript. G.L., U.D.K., L.D. and J.Z. gathered, examined, and interpreted the experimental data. X.N., G.L. and C.L. revised the manuscript for important intellectual content. W.S. and Z.Y. provided financial support for this study. The final manuscript was read and approved by all authors.

## Conflicts of Interest

The authors declare no conflicts of interest.

## Supporting information


**Figure S1.** Flow chart of this study. TCGA: The Cancer Genome Atlas. CGGA: The Chinese Glioma Genome Atlas. DEGs: Differentially Expressed genes. GO: Gene Ontology. KEGG: Kyoto Encyclopedia of Genes and Genomes. GSEA: Gene Set Enrichment Analysis. AUC: the Area Under the Curve. ROC: Receiver Operating Characteristic. DCA: Decision Curve Analysis. TME: Tumour Microenvironment. TICs: Tumour‐infiltrating Immune Cells. TISCH: Tumour Immune Single‐Cell Hub. TIMER: Tumour Immune Estimate Resources.

**Figure S2.** External validation and clinical characteristics of survival value of 10 disulfidptosis genes. A–J In CGGA database, Kaplan–Meier survival analysis of 10 disulfidptosis genes in validation set. K–L In the TCGA database, 10 disulfidptosis genes expression differences in different grade gliomas. M and N In the TCGA database, 10 disulfidptosis gene expression differences between IDH different states. O and P In the TCGA database, 10 disulfidptosis gene expression differences between 1P/19q different states. TCGA: The Cancer Genome Atlas. CGGA: The Chinese Glioma Genome Atlas. ns: no signification, *p < 0.05, **p < 0.01, ***p < 0.001.

**Figure S3.** External validation of risk score. A In the CGGA database, the risk factor map showed that the survival status of patients in the high–low‐risk assessment group was different. RPN1, NDUFA11 and GYS1 were highly expressed in the high‐risk group and NUBPL and LRPPRC were highly expressed in the low‐risk group (0: alive, 1: dead). B Kaplan–Meier survival analysis showed that high‐risk score of patient’s survival period is short. C Time ROC analysis showed that risk score 1‐, 3‐ and 5‐year OS in patients with glioma has good predictive value. D In Rembrandt database, the risk factors for figure shows differences between high and low‐risk group of patient’s condition, RPN1, NDUFA11 and GYS1 high expression in the high‐risk group, NUBPL and LRPPRC high expression in the low‐risk group (0: alive, 1: dead). E Kaplan–Meier survival analysis showed that patients with high‐risk score of short lifetimes. F Time ROC analysis showed that risk score 1‐, 3‐ and 5‐year OS in patients with glioma has good predictive value. G–I In the TCGA database, time‐dependent ROC analysis shows five disulfidptosis genes in patients with glioma 1‐, 3‐ and 5‐year OS predictive value. TCGA: The Cancer Genome Atlas. CGGA: The Chinese Glioma Genome Atlas. OS: Overall survival. ROC: Receiver Operating Characteristic.

**Figure S4.** Nomogram construction and external validation. A Nomogram containing risk score, grade and 1p/19q status was constructed in the TCGA database. B The calibration curves show Nomogram has good prediction accuracy. C In the TCGA database, build the Nomogram of the same variable. D The calibration curves show Nomogram has good prediction accuracy. E–G DCA analysis showed that nomogram net income is better than that of single variable of clinical decision‐making. TCGA: The Cancer Genome Atlas. DCA: Decision Curve Analysis.

**Figure S5.** The application value of risk score in clinical characteristics. A and B Kaplan–Meier survival analysis showed that high‐risk scores were strongly associated with shorter survival in gender subgroups. C and D Kaplan–Meier in survival analysis showed in age subgroups, high‐risk score is closely related to the survival short. E and F Kaplan–Meier survival analysis shows in 1p/19q subgroups, high‐risk score is closely related to the survival short. G Kaplan–Meier survival analysis displays in WHO II‐III group, high‐risk score group survival period is short. H The risk score increased with the increase of tumour grade. I The risk score of IDH wild group was higher than IDH mutant group; (J): Patients with 1p/19q noncodel had higher risk scores than those with codel. K The risk scores of MGMT unmethylated were higher than those of methylated patients. L Patients older than 60 years had a higher risk score than patients younger than 60 years. M There was no significant difference in risk scores between gender. ns: no signification, ***p < 0.001.

**Figure S6.** Immune therapy response between Low‐ and High‐Risk groups.

## Data Availability

TCGA, CGGA, Rembrandt, TISCH, TIMER, and CPTAC are examples of open databases. Because our analysis relies on information that is accessible to the public, there are no ethical quandaries or any other possible clashes of interest.
