# Supplementary figures and images for: Integrative Disulfidptosis‐Based Risk Assessment for Prognostic Stratification and Immune Profiling in Glioma
Source: J Cell Mol Med. 2025 Feb 24;29(4):e70429. doi: 10.1111/jcmm.70429 (PMC11850091; doi:10.1111/jcmm.70429)

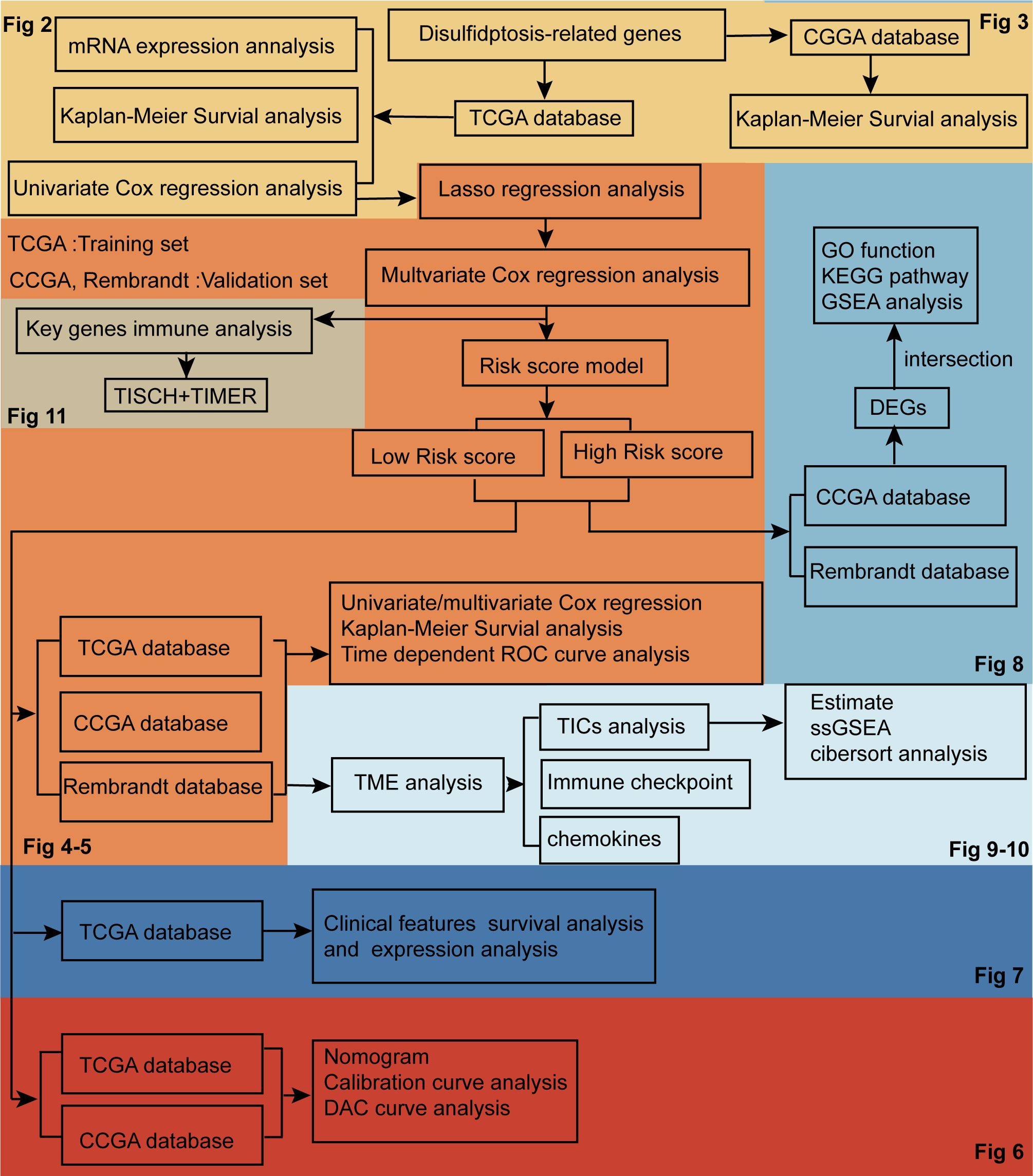

Supplement: Supplementary file 1 — Figure S1. Flow chart of this study. TCGA: The Cancer Genome Atlas. CGGA: The Chinese Glioma Genome Atlas. DEGs: Differentially Expressed genes. GO: Gene Ontology. KEGG: Kyoto Encyclopedia of Genes and Genomes. GSEA: Gene Set Enrichment Analysis. AUC: the Area Under the Curve. ROC: Receiver Operating Characteristic. DCA: Decision Curve Analysis. TME: Tumour Microenvironment. TICs: Tumour‐infiltrating Immune Cells. TISCH: Tumour Immune Single‐Cell Hub. TIMER: Tumour Immune Estimate Resources. [file JCMM-29-e70429-s001.tiff]

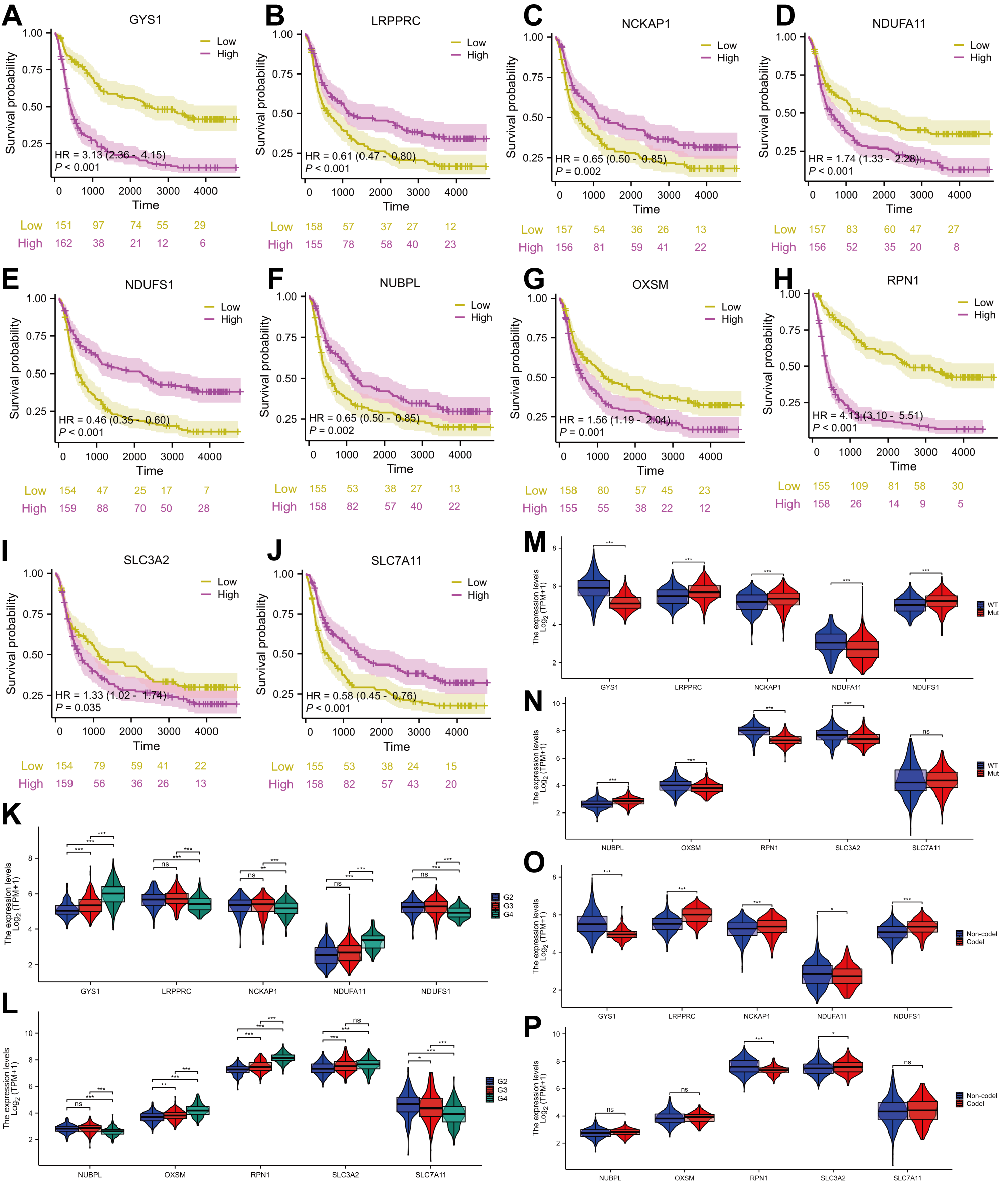

Supplement: Supplementary file 2 — Figure S2. External validation and clinical characteristics of survival value of 10 disulfidptosis genes. A–J In CGGA database, Kaplan–Meier survival analysis of 10 disulfidptosis genes in validation set. K–L In the TCGA database, 10 disulfidptosis genes expression differences in different grade gliomas. M and N In the TCGA database, 10 disulfidptosis gene expression differences between IDH different states. O and P In the TCGA database, 10 disulfidptosis gene expression differences between 1P/19q different states. TCGA: The Cancer Genome Atlas. CGGA: The Chinese Glioma Genome Atlas. ns: no signification, *p < 0.05, **p < 0.01, ***p < 0.001. [file JCMM-29-e70429-s002.tiff]

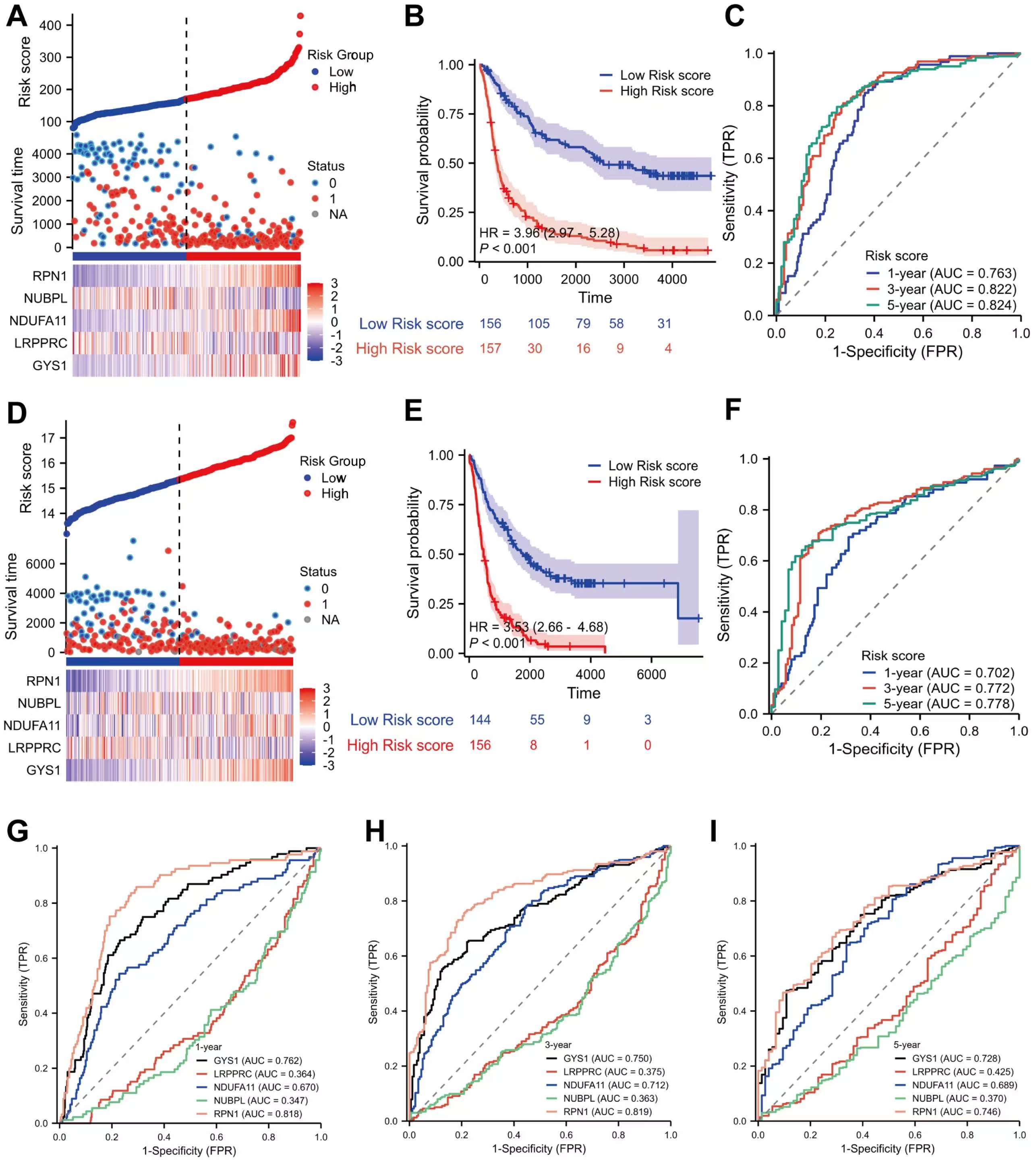

Supplement: Supplementary file 3 — Figure S3. External validation of risk score. A In the CGGA database, the risk factor map showed that the survival status of patients in the high–low‐risk assessment group was different. RPN1, NDUFA11 and GYS1 were highly expressed in the high‐risk group and NUBPL and LRPPRC were highly expressed in the low‐risk group (0: alive, 1: dead). B Kaplan–Meier survival analysis showed that high‐risk score of patient’s survival period is short. C Time ROC analysis showed that risk score 1‐, 3‐ and 5‐year OS in patients with glioma has good predictive value. D In Rembrandt database, the risk factors for figure shows differences between high and low‐risk group of patient’s condition, RPN1, NDUFA11 and GYS1 high expression in the high‐risk group, NUBPL and LRPPRC high expression in the low‐risk group (0: alive, 1: dead). E Kaplan–Meier survival analysis showed that patients with high‐risk score of short lifetimes. F Time ROC analysis showed that risk score 1‐, 3‐ and 5‐year OS in patients with glioma has good predictive value. G–I In the TCGA database, time‐dependent ROC analysis shows five disulfidptosis genes in patients with glioma 1‐, 3‐ and 5‐year OS predictive value. TCGA: The Cancer Genome Atlas. CGGA: The Chinese Glioma Genome Atlas. OS: Overall survival. ROC: Receiver Operating Characteristic. [file JCMM-29-e70429-s003.jpg]

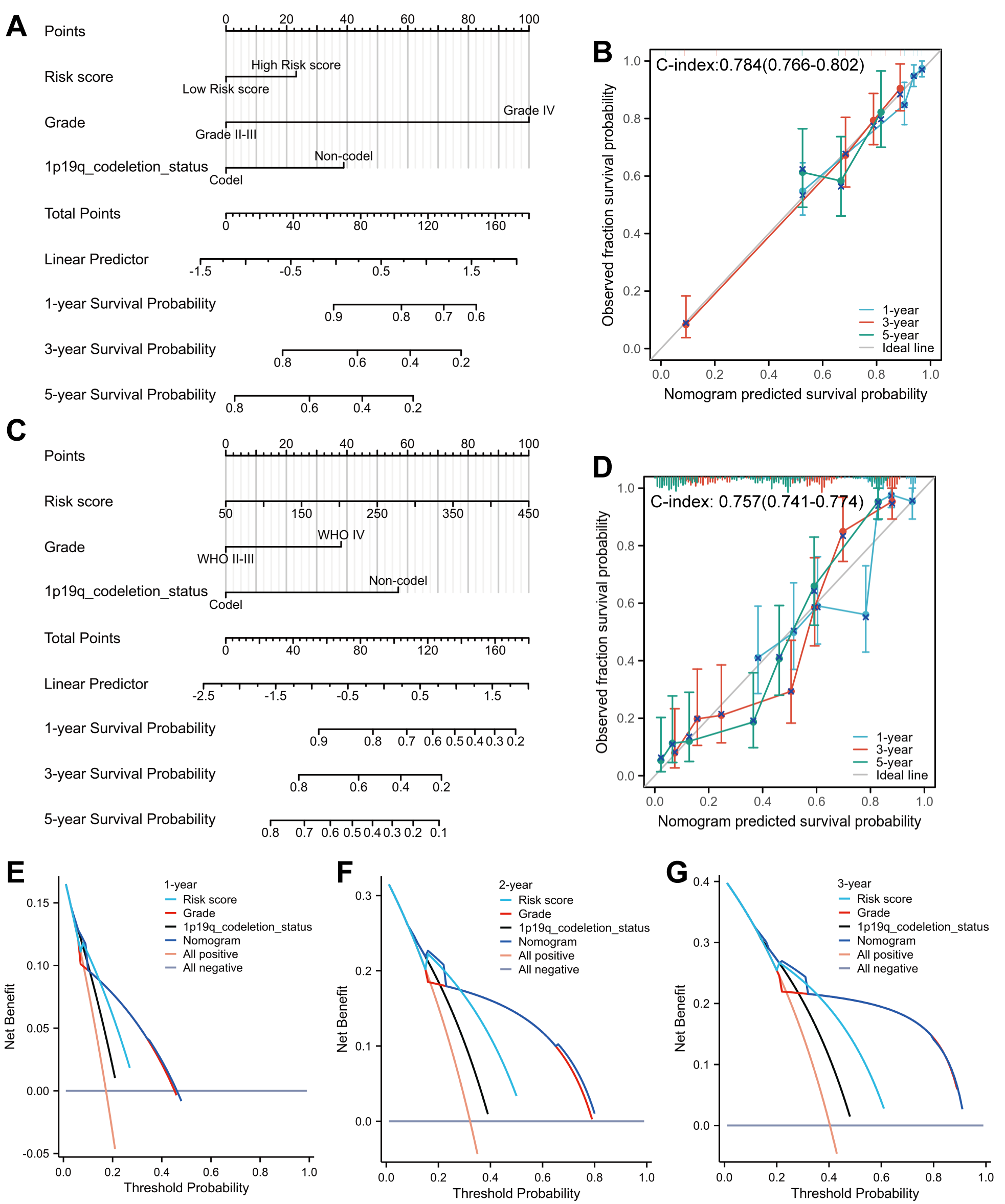

Supplement: Supplementary file 4 — Figure S4. Nomogram construction and external validation. A Nomogram containing risk score, grade and 1p/19q status was constructed in the TCGA database. B The calibration curves show Nomogram has good prediction accuracy. C In the TCGA database, build the Nomogram of the same variable. D The calibration curves show Nomogram has good prediction accuracy. E–G DCA analysis showed that nomogram net income is better than that of single variable of clinical decision‐making. TCGA: The Cancer Genome Atlas. DCA: Decision Curve Analysis. [file JCMM-29-e70429-s005.tiff]

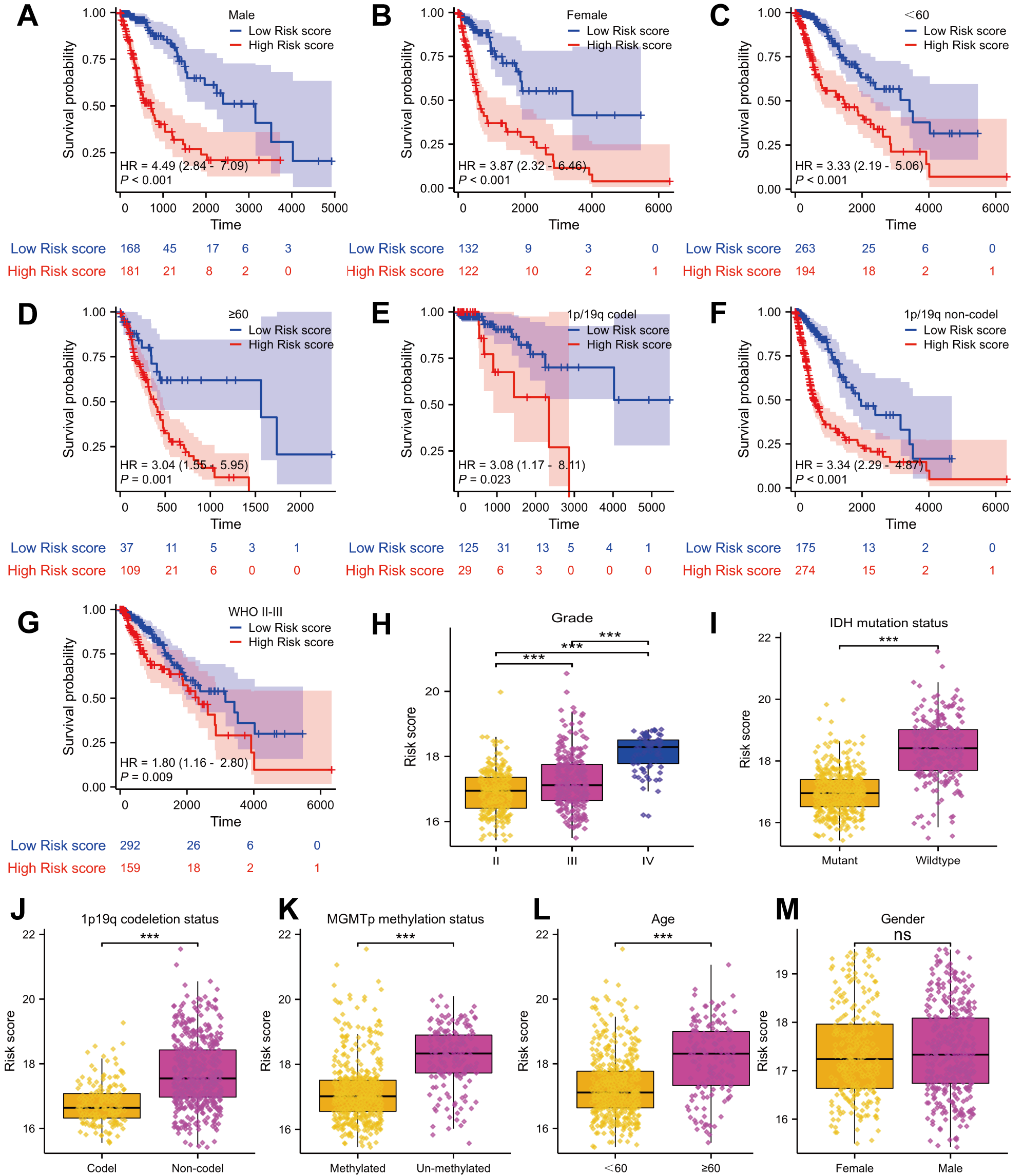

Supplement: Supplementary file 5 — Figure S5. The application value of risk score in clinical characteristics. A and B Kaplan–Meier survival analysis showed that high‐risk scores were strongly associated with shorter survival in gender subgroups. C and D Kaplan–Meier in survival analysis showed in age subgroups, high‐risk score is closely related to the survival short. E and F Kaplan–Meier survival analysis shows in 1p/19q subgroups, high‐risk score is closely related to the survival short. G Kaplan–Meier survival analysis displays in WHO II‐III group, high‐risk score group survival period is short. H The risk score increased with the increase of tumour grade. I The risk score of IDH wild group was higher than IDH mutant group; (J): Patients with 1p/19q noncodel had higher risk scores than those with codel. K The risk scores of MGMT unmethylated were higher than those of methylated patients. L Patients older than 60 years had a higher risk score than patients younger than 60 years. M There was no significant difference in risk scores between gender. ns: no signification, ***p < 0.001. [file JCMM-29-e70429-s006.tiff]

**Supplement Figure 6** Immune therapy response between Low and High Risk groups

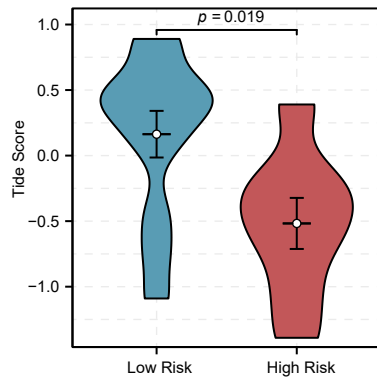

Supplement: Supplementary file 6 — Figure S6. Immune therapy response between Low‐ and High‐Risk groups. [file JCMM-29-e70429-s004.pdf]
